# Prevalence of Wēnzhōu virus in small mammals in Yunnan Province, China

**DOI:** 10.1371/journal.pntd.0007049

**Published:** 2019-02-15

**Authors:** Jinxia Wang, Xinglou Yang, Haizhou Liu, Li Wang, Jihua Zhou, Xi Han, Yan Zhu, Weihong Yang, Hong Pan, Yunzhi Zhang, Zhengli Shi

**Affiliations:** 1 Institute of Preventive Medicine, School of Public Health, Dali University, Dali, China; 2 Yunnan Provincial Key Laboratory for Zoonosis Control and Prevention, Yunnan Institute of Endemic Diseases Control and Prevention, Dali, China; 3 Key Laboratory of Special Pathogens and Biosafety, Wuhan Institute of Virology, Chinese Academy of Sciences, Wuhan, China; The University of Kansas, UNITED STATES

## Abstract

**Background:**

Mammarenaviruses are associated with human hemorrhagic fever diseases in Africa and America. Recently, a rodent mammarenavirus, Wēnzhōu virus (WENV) and related viruses, have been reported in China, Cambodia, and Thailand. Moreover, in Cambodia, these viruses were suspected to be associated with human disease. In China, Yunnan Province is famous for its abundant animal and plant diversity and is adjacent to several South-eastern Asia countries. Therefore, it is necessary to know whether WENV-related viruses, or other mammarenaviruses, are prevalent in this province.

**Methodology/Principal findings:**

Small mammals were trapped, euthanized, and sampled. Mammarenavirus RNA was detected using a nested reverse transcription polymerase chain reaction (RT-PCR) and quantified by real-time RT-PCR. A total of 1040 small mammals belonging to 13 genera and 26 species were trapped in Yunnan Province. WENV-related mammarenaviruses were detected in 41 rodent liver samples, mainly in brown rats (*Rattus norvegicus*) and oriental house rats (*R*. *tanezumi*).Viral nucleocapsid protein was detected in liver sections by indirect immunofluorescence assay. Full-length-genomes were amplified by RT-PCR and used for phylogenetic analysis with the MEGA package. Recombination analysis was performed using the SimPlot and Recombination Detection Program.

**Conclusions/Significance:**

WENV related viruses circulated in small mammals in Yunnan Province. Whole genome sequence analysis of five selected viral strains showed that these viruses are closely related to WENVs discovered in Asia and form an independent branch in the phylogenetic tree in the WENV clade. Paying attention to investigate the influence of these viruses to public health is essential in the epidemic regions.

## Author summary

Rodents are natural reservoirs of mammarenavirus. Lymphocytic choriomeningitis virus (LCMV), isolated in Asian countries during the 1990s, has a worldwide distribution and was the first mammarenavirus isolated. In 2014, a second mammarenavirus, Wēnzhōu virus (WENV), was identified in rodents in Zhejiang Province of China and later in Guangdong, Shandong, and Hainan Provinces. Most importantly, WENV or related viruses were reported in Thailand and Cambodia. In Cambodia, the isolated virus was associated with human respiratory diseases. In this study, we detected WENV or related viruses in Yunnan Province and found a high prevalence in rats of two species (*Rattus norvegicus* and *R*. *tanezumi*). Phylogenetic analysis of the complete L and S segments of five strains showed that these viruses form an independent phylogenetic branch in WENV clade most closely related to WENVs found in China and Cambodia. Considering the wide spread distribution of rats and altered distribution patterns due to ecological changes, we propose that these viruses may have a wider prevalence and be found in countries from South-eastern Asia to China. Given that WENV may be associated with human diseases, it is necessary to improve surveillances of these viruses in their natural reservoirs and in humans.

## Introduction

Mammarenaviruses, belonging to the genus *Mammarenavirus*, family *Arenaviridae*, are associated with several hemorrhagic fever diseases around the world [1]. Mammarenavirus particles are spherical to pleomorphic in shape and, range from 40 to 300 nm in diameter [2]. The virus particle carries a variable number of ribosomes (20–25 nm) from host cells that are responsible for their sandy, or arena-like, appearance under electron microscopy[3]. Mammarenavirus possess a bi-segmented, ambisense, single-stranded RNA genome. The L and S segments share conserved sequences at both ends and are around 7.2 and 3.5 kb long, respectively [4]. Each genomic RNA encodes two different proteins in opposite orientations [5]. The two open reading frames (ORFs) in each segment are separated by an intergenic noncoding region that forms one or more energetically stable stem-loop (hairpin) structures [6]. The L segment encodes a viral RNA-dependent RNA polymerase (RdRp, L) and a zinc binding matrix protein (Z). The S segment encodes a nucleoprotein (NP) and an envelope glycoprotein precursor (GPC) [2].

Rodents are the primary reservoir of mammarenaviruses, with the exception of the Tacaribe virus that was isolated from Jamaican fruit-eating bats (*Artibeus jamaicensis*) and Wēnzhōu virus (WENV) also detected in shrews [7–9]. Most mammarenaviruses induce a persistent and frequently asymptomatic infection in their reservoir hosts and are associated with a specific host species or group of species [10]. Mammarenaviruses can be transmitted through rodent urine or blood [11]. Humans usually become infected through contact with infected rodents or inhalation of infectious rodent excreta or secretions [2]. Animal models, such as hamsters, mice, guinea pigs, and non-human primates, have been used to study the pathogenicity of mammarenaviruses and to measure vaccine or drug efficacy [12]. Viral infections have been identified in many organs, including the spleen, liver, adrenal gland, placenta, and lungs [10, 13–17].

The *Mammarenavirus* genus is divided into the two main groups corresponding to Old and New World viruses. Mammarenaviru*s* infections contribute significantly to the human disease burden in both Africa and the Americas, but little is known about the disease burden in Asia [18]. Lassa virus (LASV) causes Lassa hemorrhagic fever (LHF) with a mortality of between 5,000 and 10,000 each year in West Africa[19, 20], whilst Junín virus (JUNV), Machupo virus, Sabía virus, and Guanarito virus cause fatal hemorrhagic fever diseases and had a mortality rate between 20 and 30% in Latin America[18, 21]. In 2014, a rodent arenavirus, WENV was identified in Wēnzhōu, Zhejiang Province, China [8]. In 2015, a genetic variant of WENV, associated with influenza-like illness, was found in Cambodia and Thailand [22]. Recently, similar virus sequences were reported in Shandong, Guangdong, and Hainan provinces, China [9, 23–26].

Yunnan Province is notable for its plant and animal diversity and is geographically adjacent to South-eastern Asian countries. Diverse hantaviruses, coronaviruses, hepatitis viruses, paramyxoviruses, and astroviruses had been reported in rodents in Yunnan Province[27–33]. There are more than 88 rodent species belonging to eight families in Yunnan Province [34].Therefore, it is essential to study the distribution of mammarenaviruses in Yunnan Province to understand its genetic evolution and geographic distribution.

## Materials and methods

### Ethics statement

The collection of small wild animals was performed by veterinarians with approval from the Animal Ethics Committee of Yunnan Institute of Endemic Diseases Control and Prevention (Animal ethics approval number: 201302).

### Sampling

**Fig 1:**
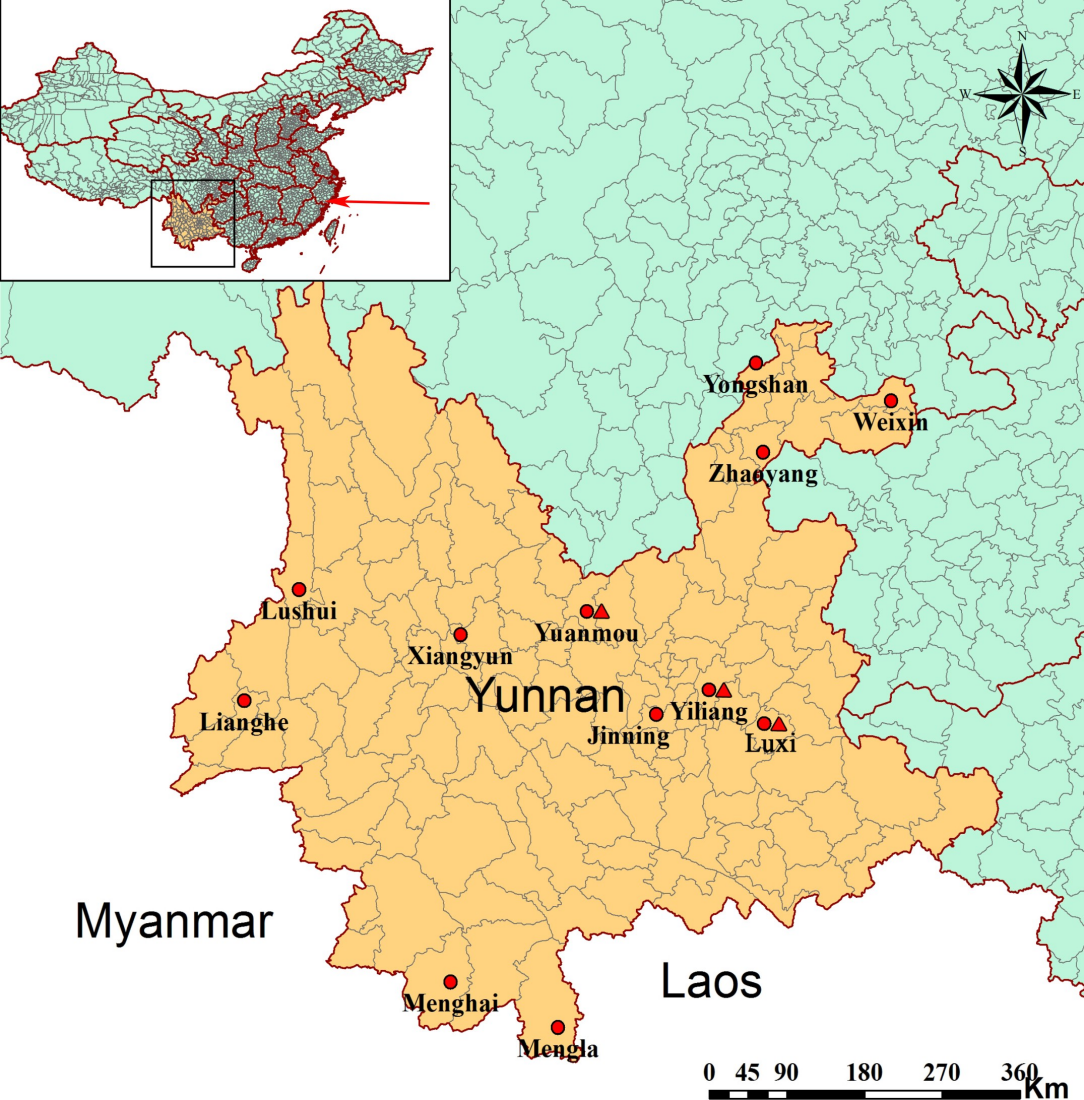
Map showing the small mammal collection sites in Yunnan Province of China. Sampling map drawn using QGIS Desktop 3.0.1 software. Red circles indicate sample collection sites, while red triangles indicate the sites where RNA virus was detected. The red arrow in the inset map indicates the location (Wēnzhōu city) where WENV was reported for the first time.

From April 2015 to September 2017, small mammals were trapped in 12 counties in urban or residential districts of eastern and western Yunnan Province (Fig 1). Small animals were captured through mouse traps using fried foods as bait. Animals were assigned to species through morphologic observation and confirmed by mitochondrial cytochrome B DNA amplification and sequencing. Captured animals were humanely euthanized and blood and/or organs (heart, liver, spleen, lung, kidney, and intestine) were collected, stored temporarily in liquid nitrogen, and transferred to the laboratory where they were stored at −80ºC until use.

### RNA extraction and virus detection

Animal tissues were thawed and viral RNA extracted from approximately 0.1 g of tissue using the High Pure Viral RNA Kit (Roche, Germany). A nested reverse transcription polymerase chain reaction (RT-PCR) was performed using degenerate primers targeting the conserved RdRp regions, as previously described [8, 9]. In brief, the first round of PCR was performed in a 25 μl reaction mix using the SuperScript III/ Platinum Taq Enzyme Mix, and 3 μl of RNA as a template. The second round of PCR was performed using the Platinum Taq Enzyme (Invitrogen, USA), and 1 μl of the first-round PCR product as a template. The resultant amplicons were separated by agarose gel electrophoresis and bands of the expected sizes were gel purified and directly sequenced or cloned into the pGEM-T Easy Vector (Promega, USA) if direct sequencing was unsuccessful.

### Quantitative real-time RT-PCR (qRT-PCR)

A qRT-PCR using the HiScript II One Step qRT-PCR SYBR Green kit (Vazyme Biotech, Nanjing, China) was developed and optimized according to the manufacturer’s instructions using 20 μl reaction volumes. Specific primers (5′-AGAAGGAAGATGCTCTTGTT-3′ and 5′-AAGACCTGATTGAGTGTTGG-3′) were designed based on the sequence obtained in this study. A plasmid containing the target sequence was used for viral RNA transcription *in vitro* and for generating the standard curves for viral RNA quantification. PCR conditions were as follows: 3 min at 50°C for reverse transcription; 5 min at 95°C for activation of Taq DNA polymerase; and 40 cycles at 95°C for 10 sec and 60°C for 30 sec. Positive samples were characterized by a well-defined exponential fluorescence curve that crossed the cycle threshold (C_t_) within 36 cycles. Specimens with a C_t_ >36 were considered negative. Each run included three viral positive template controls and two negative controls to monitor performance. Viral genome copies were calculated in each sample using the standard curves of the template RNA.

### Sequencing of full-length genomes

Viral genomes of the detected rodent mammarenavirus were amplified by nested-PCR and rapid amplification of cDNA ends (RACE). In brief, liver sample RNA was reverse transcribed to cDNA using MLV reverse transcriptase (Promega, USA) then eight short fragments were amplified using eight primer pairs (sequences provided upon request) based on the conserved domains of the L and S fragment sequences. The remaining gaps were filled by specific primers that were designed based on the obtained sequences. Genomic 5′ and 3′ ends were amplified using the SMARTer RACE cDNA Amplification Kit (Clontech, USA). Each PCR product was cloned into the pGEM-T Easy Vector (Promega, USA) and at least three clones for each PCR fragment were sequenced to obtain a consensus sequence. Sequences were assembled by Geneious R10 software (Biomatters, New Zealand).

### Phylogenetic analysis

Genomic nucleotide (nt) sequences and the deduced ORF amino acid (aa) sequences were compared to those of representative mammarenaviruses using Geneious R10 software (Biomatters, New Zealand). Recombination events were scanned for the sequences of Yunnan isolates using the Recombination Detection Program [35]. Suggested recombination events with strong p value were further confirmed in Simplot 3.5.1 [36]. Phylogenetic trees were constructed using neighbor-joining methods from MEGA 7 [37].

### Virus isolation

Virus isolation was conducted using canine macrophage cell DH82 and VeroE6 as described previously [8]. Cells were cultured and inoculated with viral RNA positive samples after a 10-fold dilution. The inoculated cells were incubated in the Dulbecco's Modified Eagle's Medium (DMEM) with 2% fetal bovine serum (FBS). After three blind passages, the cell culture supernatant was tested for the presence of virus using the nested RT-PCR.

### Mammarenavirus antibodies detection

The enzyme linked immunosorbent assay (ELISA) for mammarenavirus antibody detection was developed based on a procedure of a previous study [38]. In brief, the *NP* gene was amplified and cloned into a pET expression plasmid fused with a C-terminal His tag. The NP-His fusion was expressed in *E*. *coli* BL21 (DE3) cells and purified by Profinity IMAC Nickel Charged Resin (BIO-RAD, US) according to the manufacturer’s instructions. Purified NP was used to immunize rabbits to obtain rabbit polyclonal antibodies. Rodent serum samples were diluted 1:20 and added to the ELISA plate coated with NP. The Peroxidase conjugated Affinipure Goat Anti Mouse antibodies (Proteintech, Wuhan, China) were used as the secondary antibodies. Absorbance values that were three times greater than those of the negative control were considered positive. For western blotting, 200 ng of NP was separated by sodium dodecyl sulfate polyacrylamide gel electrophoresis and transferred onto a polyvinylidene difluoride membrane. Rodent serum samples were diluted (1:100) and the peroxidase conjugated Affinipure Goat Anti Mouse (H+L) (Proteintech, Wuhan China) used as secondary antibody. The His-tagged human coronavirus NL63 N was used as parallel control.

### Hematoxylin eosin (HE) staining and indirect immunofluorescence (IFA)

Liver tissues from rodents positive or negative for mammarenavirus RNA were fixed in 10% formalin for 36 h. Samples were then dehydrated, paraffin embedded, sectioned, and mounted onto glass slides. After staining with HE, the slides were examined using microscopy (Olympus, Japan). Sections (5 mm) of formalin-fixed paraffin-embedded tissues were placed onto positively charged glass slides and air dried for 30 min. The tissue sections were deparaffinized, rinsed, and incubated with target retrieval solution (Sigma-Aldrich, USA). After the sections were blocked with 5% FBS (Gibco, Australia), they were incubated with anti-NP rabbit serum for 30 min at 37°C followed by incubation with goat anti-rabbit IgG cyanine 3 (Cy3)-conjugated secondary antibody (TransGen Biotech, China). The sections were checked using an immunofluorescence laser scanning confocal microscope (Zeiss, Germany).

## Results

### Prevalence of mammarenaviruses in small mammals

A total of 1040 small mammals, belonging to 13 genera and 26 species, were trapped in 12 counties (Fig 1 and Table 1). A total of 41/1040 (3.94%) liver tissue samples were positive for mammarenavirus RNA, including 34/195 (17.44%) brown rat samples, 5/328 (1.53%) oriental house rat samples, 1/12 (8.33%) Himalayan field rat (*R*. *nitidus*) samples, and 1/10 (10%) Northern tree shrew (*Tupaia belangeri*) sample. Among the 41 positive samples, 16 brown rat samples were from Luxi County, three brown rat samples were from Yiliang County, and all other samples were collected from Yuanmou County.

**Table 1:** PCR detection results for Arenavirus in small mammals collected in Yunnan Province during 2015 to 2017.

Family	Genus	Species	Common name	Sampling site(s) with positive(P/D *; yr)	Sampling site(s) with negative(samples tested; yr)	P/D *
Cricetidae	Eothenomys	*E*. *eleusis*	Small Oriental Vole		Lushui(15; 2015),Xiangyun(1; 2015),Yongshan(2; 2015), Zhaoyang(1; 2016), Yuanmou(36; 2017).	0/55
		*E*.*miletus*	Large Oriental Vole		Xiangyun(17;2015).	0/17
Muridae	Apodemus	*A*. *agrarius*	Striped Field Mouse		Weixin(3; 2016).	0/3
		*A*.*chevrieri*	Chevrier's Field Mouse		Jining(9; 2015), Lushui(33; 2015), Xiangyun(57; 2015–2017), Yuanmou(41; 2015–2017), Lianghe(7; 2016), Weixin(19; 2016), Yiliang(18; 2016), Luxi(82; 2016–2017).	0/266
		*A*. *draco*	South China Field Mouse		Lushui(4; 2015), Menghai(1; 2016), Yuanmou(3; 2016).	0/8
	Bandicota	*B*.*indica*	Greater Bandicoot Rat		Lianghe(1; 2016).	0/1
	Mus	*M*. *caroli*	Ryukyu Mouse		Xiangyun(1; 2015), Yuanmou(2; 2015–2016), Lianghe(1; 2016), Yiliang(13; 2016).	0/17
		*M*.*musculus*	House Mouse		Mengla(1; 2016), Weixin(3; 2016), Zhaoyang(4; 2016).	0/8
		*M*. *pahari*	Indochinese Shrewlike Mouse		Xiangyun(2; 2015), Yongshan(1; 2015), Lianghe(1; 2016), Yuanmou(1; 2016), Yiliang(5; 2016).	0/10
	Niviventer	*N*. *eha*	Smoke-bellied rat		Lushui(6; 2015).	0/6
		*N*. *fulvescens*	Indomalayan Niviventer		Lianghe(2; 2016), Yiliang(2; 2016).	0/4
		*N*.*niviventer*	Himalayan Niviventer		Lushui(1; 2015).	0/1
	Rattus	*R*.*andamanensis*	Indochinese Forest Rat		Yuanmou(5; 2015), Lianghe(10; 2016).	0/15
		*R*.*nitidus*	White-footed Indochinese Rat	Yuanmou(1/6; 2015)	Menghai(1; 2016), Weixin(4; 2016), Yiliang(1; 2016).	1/12
		*R*.*norvegicus*	Brown Rat	Yuanmou(15/92; 2015–2017), Yiliang(3/3; 2016), Luxi(16/82; 2017)	Jining(2; 2015), Xiangyun(10; 2015), Yongshan(1; 2015), Weixin(5; 2016).	34/195
		*R*.*rattus*	Roof Rat		Yuanmou(1; 2015), Lianghe(1; 2016), Menghai(8; 2016).	0/10
		*R*. *tanezumi*	Oriental House Rat	Yuanmou(5/34; 2015- 2017),	Jining(8; 2015), Xiangyun(2; 2015). Weixin(19; 2016), Mengla(30; 2016), Lianghe(229; 2016), Luxi(6; 2017).	5/328
Mustelidae	Mustela	*M*.*sibirica*	Siberian weasel		Yuanmou(1; 2015).	0/1
Soricidae	Anourosorex	*A*. *squamipes*	Chinese Mole Shrew		Lushui(1; 2015),.Weixin(24; 2016), Yuanmou(1; 2017).	0/26
	Chodsigoa	*C*. *salenskii*	Salenski's Shrew		Lushui(4; 2015).	0/4
	Crocidura	*C*. *attenuata*	Asian Gray Shrew		Yuanmou(10; 2015–2017), Lianghe(7; 2016), Weixin(1; 2016).	0/18
	Sorex	*S*.*alpinus*	Alpine Shrew		Lushui(9; 2015).	0/9
		*S*. *bedfordiae*	Lesser Striped Shrew		Lushui(5; 2015), Yongshan(1; 2015).	0/6
	Suncus	*S*. *murinus*	Asian House Shrew		Lianghe(6; 2016), Menghai(2; 2016), Mengla(2; 2016).	0/10
Tupaiidae	Tupaia	*T*.*belangeri*	Northern Treeshrew	Yuanmou(1/7; 2015–2016),	Jining(1; 2015),Xiangyun(1; 2015), Luxi(1; 2017).	1/10
Total						41/1040

*, D, P, positive number(s); detected sample number(s).

**Fig 2:**
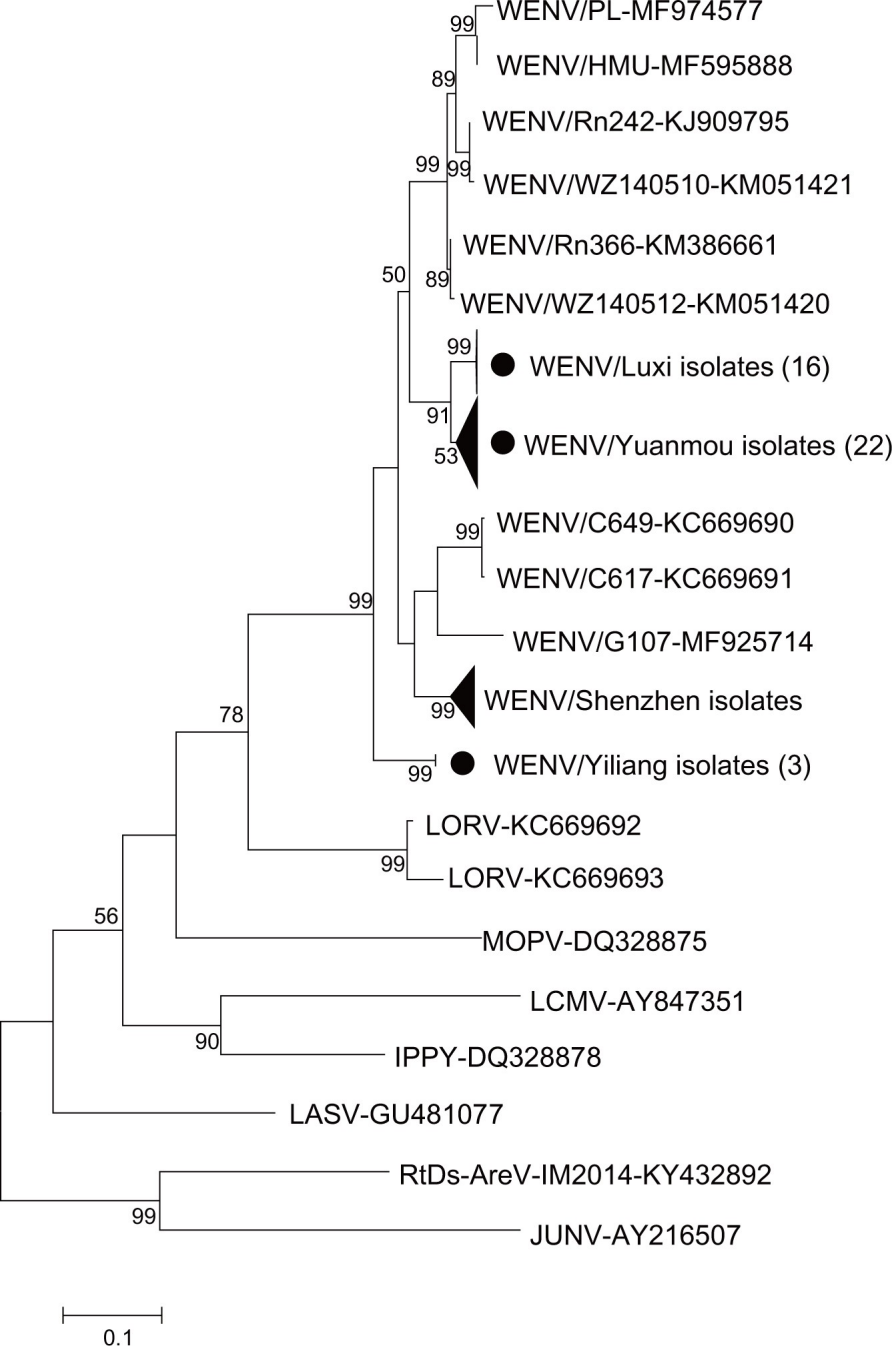
The phylogenetic tree based on partial RdRp sequences detected. The neighbor-joining tree was based on 600 nt L segment sequences and constructed using MEGA 7 software. Bootstrap values were 1,000. The scale bar indicates nucleotide substitutions per site. New sequences obtained in this study are indicated with black circles and the number in the brackets represents positive sequence numbers.

The 41 partial viral sequences (600 bp) share 85–100% similarity with each other. Furthermore, the isolated viral sequences share 86–91% similarity with the closest strain discovered in Wēnzhōu, which is higher than the similarity observed with Cambodian isolates (86–88%) and Thai isolates (70–73%). These results suggest that viral sequences detected at the same location are closely related irrespective of host differences. The phylogenetic tree shows that the viruses detected in Yunnan Province form an independent lineage that is divided into two branches, one containing the sequences from Yuanmou and Luxi counties, and the other containing the sequences from Yiliang County (Fig 2).

### Genome characterization

Because the failure of virus isolation, five liver samples were selected for full length virus genome, including two each from oriental house rats (WENV/Rt2015YM16 and WENV/Rt2015YM51) and brown rats in Yuanmou (WENV/Rn2016YM03 and WENV/Rn2016YM51) and one from brown rat in Yiliang (WENV/Rn2016YL04). All virus and partial host cytochrome b gene sequences obtained in this study have been submitted to GenBank (MG736216-MG736236; MK192269-MK192293; MK177225-MK177229).

The lengths of the L and S segments ranged from 7140–7153 bp and 3318–3377 bp, respectively. Highly conserved non-coding regions were identified in both segments (25 and 55 for the L and S segments, respectively). All L segments contained two ORFs which encoded a RdRp of 2,223 amino acids and a Z of 92 amino acids, either side of an 124 nucleotide non-coding sequence. All S segments contained two ORFs which encoded an NP of 568 amino acids and a GPC of 493 amino acids, either side of a 62 nucleotide non-coding region.

Nucleotide and deduced ORF amino acid sequences from the five isolates were compared to those of representative mammarenaviruses (Table 2). Similar to results obtained from the analysis of the partial sequences, viral strains detected in the same Yunnan Province location are most closely related, irrespective of their host species. The most closely related strains are the Zhejiang and Cambodian viriants of WENV, with L and the S segment nucleotide identities of 85.4–87.0% and 83.0–87.7%, respectively. Based on the International Taxonomy Committee on Viruses (ICTV) *Arenaviridae* Study Group guidelines for new mammarenavirus species, the Yunnan strains examined here are strain of WENV (species *Wenzhou mammarenavirus*)[1, 39]

**Table 2:** Genome or ORF comparisons between various viriants of Wēnzhōu virus and other mammarenaviruses.

Isolates	Segment/ORF	nt/aa	WENV/Yiliang	WENV/ Zhejiang	WENV/ Cambodia	LORV	LASV	JEAreV	IPPYV	MOPV	LCMV	JUNV
WENV/ Yuanmou	L segment	nt	86.7–87.0	85.1–87.2	86.4–87.0	69.3–69.7	61.6–62.0	50.6–50.9	60.5–60.7	62.5–62.6	58.2–58.6	51.8–52.0
	RdRP	nt	84.1–84.3	84.0–84.9	84.1–84.7	64.4–64.9	56.8–57.0	53.2–53.5	57.0–57.2	56.9–57.1	53.6–53.9	47.7–47.9
		aa	89.8–90.0	89.8–90.4	90.0–90.4	36.6–36.9	54.5–54.7	44.2	56.1–56.3	54.8–55.1	47.7–47.8	66.8–67.2
	Z ORF	nt	82.6–84.1	84.8–89.3	83.3–85.5	64.9–67.0	56.3–58.5	54.7–57.2	57.5–59.3	58.7–60.9	53.8–55.3	46.8–48.3
		aa	83.5–87.9	90.4–93.9	89.5–93.9	73.7–77.2	58.8–61.4	52.7–54.9	60.5–62.3	57.0–58.8	59.6	43.0–44.7
	S segment	nt	81.7–92.3	81.8–88.3	85.3–87.6	70.8–74.4	67.8–69.3	55.2–55.7	68.2–70.0	67.2–69.0	61.4–63.3	53.5–55.3
	NP ORF	nt	86.7–97.1	87.1–88.1	87.3–87.6	73.3–74.2	66.7–67.6	59.9–60.5	69.4–70.4	67.9–68.7	63.5–64.1	57.8–58.0
		aa	95.3–99.5	96.1–96.5	73.4–73.6	96.0–96.3	73.7–73.9	5937–60.6	74.2–74.4	65.0–65.5	51.5–52.2	82.2–83.7
	GPS	nt	86.2–97.6	83.4–87.6	85.0–86.9	70.5–71.7	67.2–67.9	56.4–57.1	65.6–66.3	65.6–66.3	58.7–59.4	51.2–51.9
		aa	93.1–98.1	88.8–94.3	93.0–94.1	79.4–80.4	73.9–74.9	53.8–54.6	68.4–69.7	72.0–72.8	55.8–56.4	40.0–40.8
WENV/ Yiliang	L segment	nt	-	85–86.2	87	69.6–69.7	64.7	50.5	61.1	62.7	58.7	52.0
	L ORF	nt	-	84.2–84.5	84.7–84.8	64.9	56.6	53.1	58.1	57.2	53.8	47.9
		aa	-	90.2–90.8	90.5–90.6	67.5–67.7	55.0	44.0	56.6	54.5	47.9	36.6
	Z ORF	nt	-	80.6–82.6	84.8–85.1	67.8–68.1	58.5	57.9	58.2	62.0	56.0	48.7
		aa	-	89.9	91.7	73.4	54.1	51.6	58.7	56.0	61.5	42.2
	S segment	nt	-	81.3–84.0	63.1–82.7	67.7–70.0	48.4	55.7	66.1	65.0	57.9	73.1
	NP ORF	nt	-	88.3	87.8–87.9	73.4–74.3	66.4	59.8	69.2	68.1	63.9	58.1
		aa	-	96.1–96.5	95.5–95.6	83.3–83.8	73.6	60.6	74.2	74.4	65.0	51.5
	GPS	nt	-	84–87.5	85.3–85.5	70.8–71.5	67.0	57.2	65.8	66.2	58.8	52.1
		aa	-	91.2–95.0	93.3–93.7	80.2–80.6	73.3	53.8	69.1	72.4	56.0	40.6

Note where (L & S gene): WENV/Cambodia = KC669690, KC669691& KC669694, KC669696; LORV = KC669692, KC669693 & KC669697, KC669698; WENV/zhengjiang = KM386661, KM051421, KJ909795, KM051420, MF595888, MF974577, MF925714 and KM386660, KM051423, KJ909794, KM051422, MF595889, MF97478, MF925715; LASV = GU481076 & GU481077; JEAreV = KY432892 & KY432893; IPPYV = DQ328877 & DQ328878; MOPV = DQ328874 & DQ328875; LCMV = AY847350 & AY847351; JUNV = D10072 & AY216507; ORF = Open reading frame; NP = Nucleoprotein; GPC = Glycoprotein; nt = Nucleotide; aa = Amino acid; LCMV = lymphocytic choriomeningitis Virus.

### Phylogenetic and recombination analysis of viral genomes

**Fig 3:**
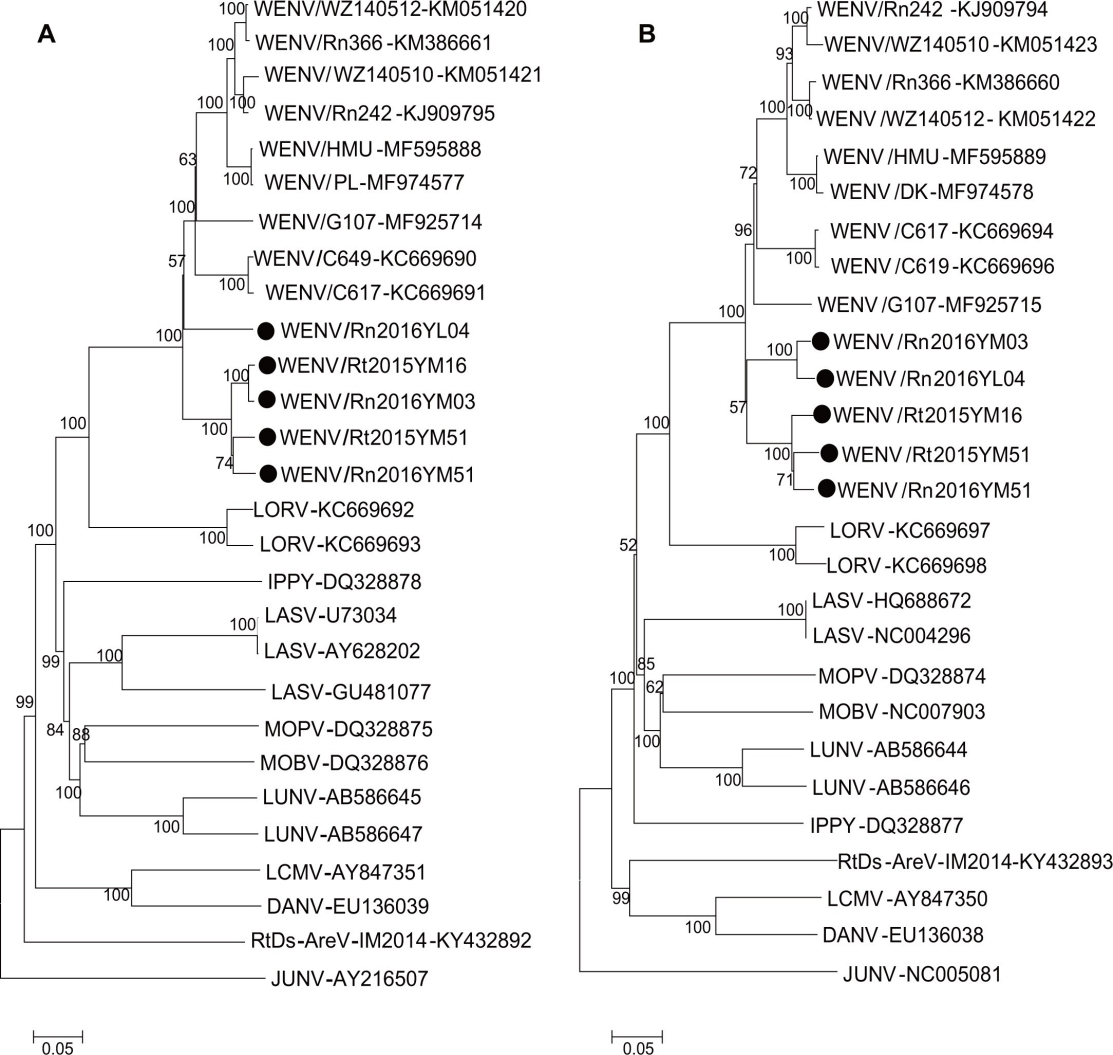
Phylogenetic analysis of mammarenavirus genomes obtained in this study. The neighbor-joining tree was constructed with MEGA 7 software based on the alignment of complete L segment (A) and S segment (B) sequences of mammarenaviruses detected in this study, and representative mammarenaviruses. Bootstrap values were 1,000. New genome sequences obtained in this study are indicated with a black circle.

**Fig 4:**
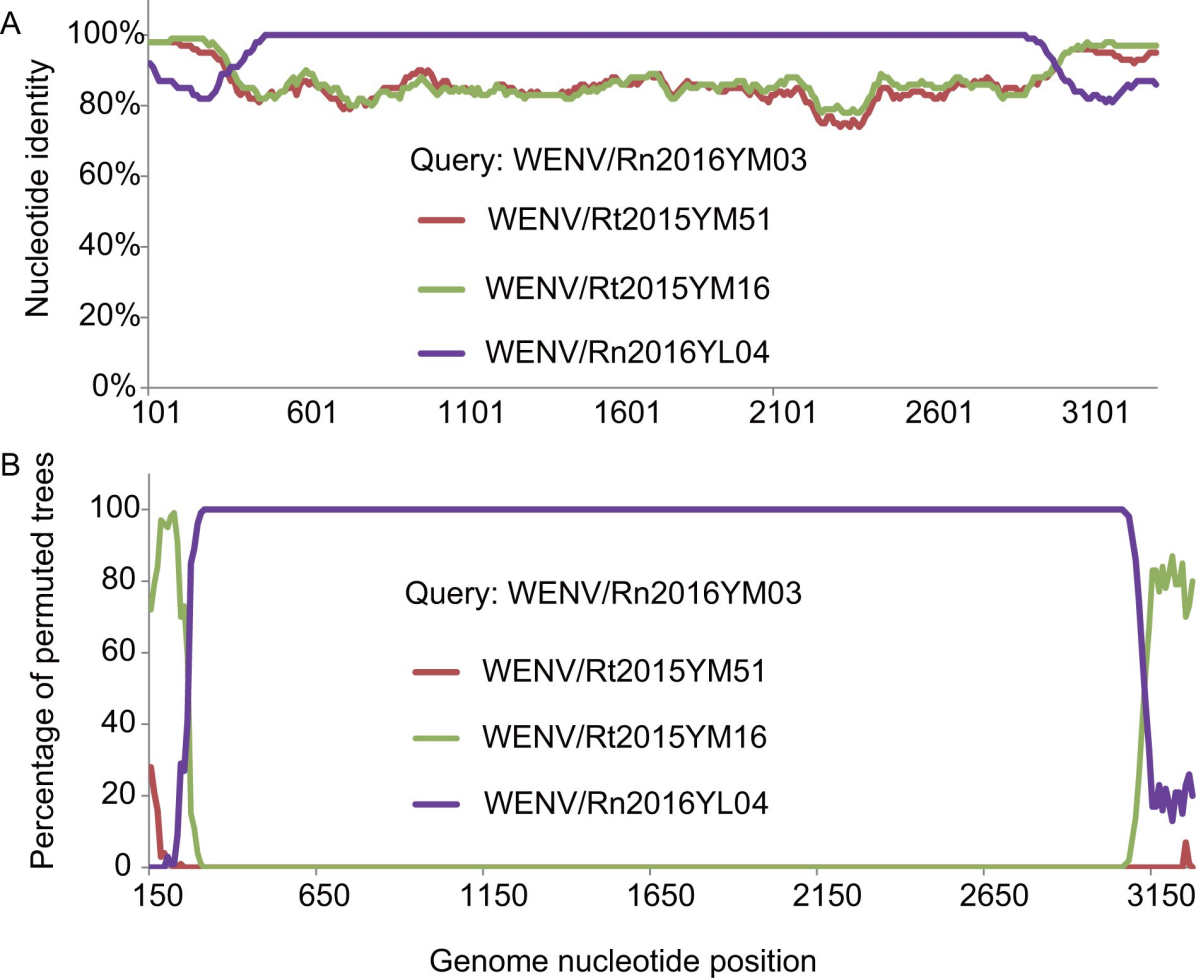
Recombination events detected by similarity plot and boot scan analysis. (A) The similarity of WENV/Rn2016YM03 and Rt2015YM03, Rt2015YM16, and Rn2016YL04 S segment sequences. (B) The recombination events detected by boot scan, WENV/Rn2016YM03 is included as a reference sequence. Analyses were performed using a Kimura model, a window size of 300 base pairs, and a step size of 20 base pairs.

Phylogenetic analysis of complete L segments showed that mammarenaviruses detected in South-eastern Asia form a distinct lineage in which the viruses are geographically separated (Fig 3A). The topology of the tree changed for the S segments of Yunnan isolates, indicating potential genetic reassortments between the detected viruses in this study (Fig 3B). After recombination scanning, we found an obvious recombination event in the S segment of WENV/Rn2016YM03 with strong p value (< 10^−38^), indicating that WENV/Rn2016YM03 is likely a result of recombination between WENV/Rn2016YL04 and WENV/Rt2015YM16 (Fig 4).

### Quantification of mammarenaviruses in different tissues by real-time PCR

**Fig 5:**
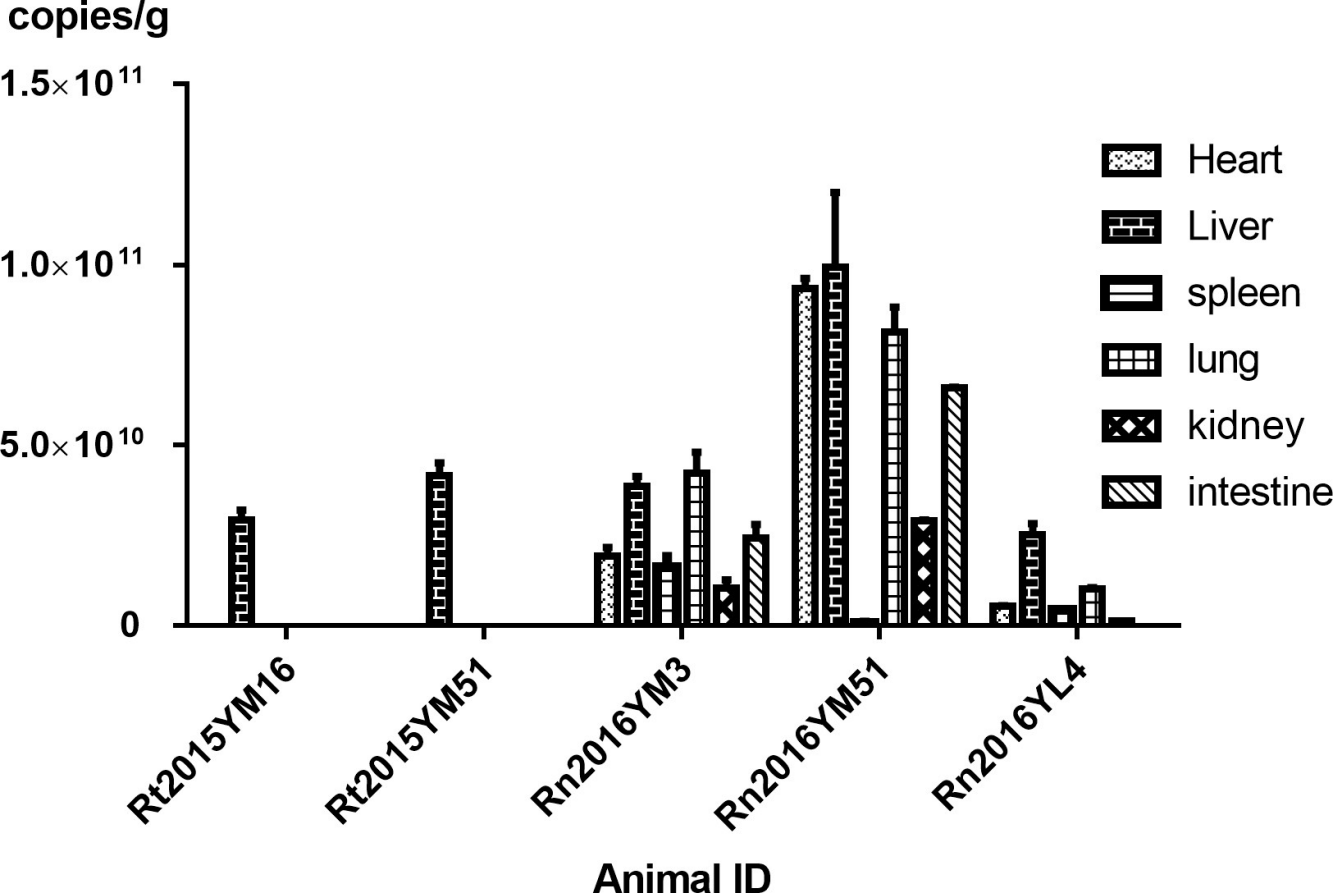
Real-time PCR quantification of viral genomic RNA. Five positive samples were selected and real-time PCR was used to quantify the viral RNA in the heart, liver, spleen, lung, kidney, and intestine (mean ± standard error of the mean). Units are presented in copies per gram.

To determine the tropism of these mammarenaviruses in their natural hosts, viral genomic RNA was quantified in tissue samples (heart, liver, spleen, lung, spleen, and intestine) by qRT-PCR. The results showed that the viral load in the positive samples ranged from 5.1 ×10^8^ to 1.14 × 10^11^ copies per gram of tissue (Fig 5). Viral RNA was detected in liver tissues of all five positive samples. Some viruses showed wider tissue tropism including the heart, spleen, lung, kidney, and intestine. The highest viral load was found in samples collected in Yuanmou in 2016.

### Virus protein detection and histopathology in rodent liver tissues

**Fig 6:**
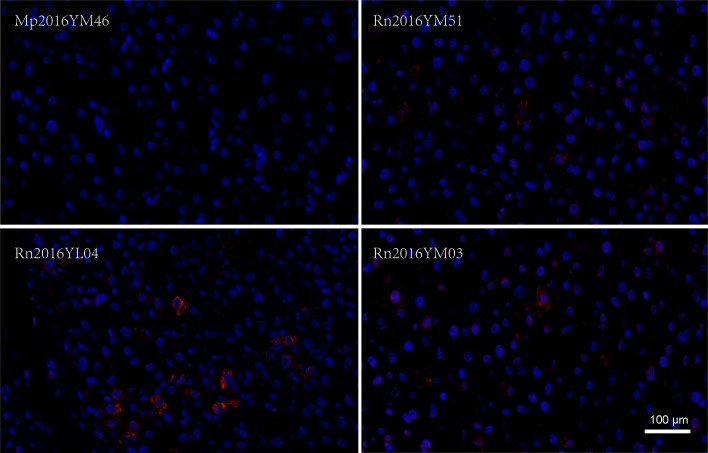
Viral protein detection by indirect immunofluorescence in liver sections. The red color showed the presence of viral NP. The blue nucleus was stained by 4′, 6-diamidino-2-phenylindole (DAPI). Three WENV positive liver samples (Rn2016YM51, Rn2016YL04, and Rn2016YM03) and one negative (Mp2016YM46) sample were selected for testing by indirect immunofluorescence. Bar represents 100 μm.

The histopathology of the virus was examined in liver sections in both viral RNA-positive and viral RNA-negative animals (*M*. *pahari*). Although indirect immunofluorescence, using rabbit polyclonal antibodies against viral NP detected virus infection, no obvious lesions were observed in viral positive livers (Fig 6).

### Seroprevalence of mammarenavirus in rodent serum samples

The cutoff absorbance value for positive serum samples was set at 0.036, a value three times greater than that of the negative control. Overall, 24 of 118 serum samples were seropositive to NP (24/118, 20.34%) (S1 and S2 Figs and S1 Table). All positive serum samples were from rodent species in which the viral nucleic acid had been detected.

## Discussion

In this study, we conducted an investigation of WENV infections in small mammals in a wide geographical area of Yunnan Province, China. Our results demonstrated, for the first time, that WENV is prevalent in three of five *Rattus* species and one *Tupaia* species in three geographically related counties. Together with previous reports, we have shown that WENV or related viruses have a host range that includes rats (*R*. *norvegicus*, *R*. *rattus*, *R*. *losea*, *R*. *exulans*, *R*. *tanezumi*, and *R*. *nitidus*), White-bellied Rat (*Niviventer niviventer*), Asian house shrews (*Suncus murinus*), and Tree shrews (*T*. *belangeri*) [8, 9, 22, 24–26]. No mammarenavirus was detected in other locations, suggesting that the distribution of these viruses may currently be limited to these three regions. However, considering the movement of rodents due to ecological changes, we cannot exclude the possibility of these viruses spreading in the future. Therefore, it is necessary to continue surveillance for these viruses in their natural reservoirs.

Sequences analysis of five full-length genomes showed that these discovered viruses are in fact closely related to WENVs, which indicated a common ancestor. But the diversity within that clade also revealed an independent lineage of Asian arenaviruses[8, 9, 22, 24, 25]. The phylogenetic tree based on partial L sequences had different shape with the tree based on complete length of L segment suggesting more complexity evolution of WENV. Meanwhile, the tree topologies for the L and S segments of the Yunnan isolates are also incongruent. In the L tree, the Yunnan strains are separated according to the two sampling locations irrespective of host species, while two strains from different locations are closely related in the S tree (Fig 3). Recombination analysis suggested that one of the Yunnan strains, WENV/Rn2016YM03, results from an S segment recombination event between WENV/Rn2016YL04 and WENV/Rt2015YM16. We hypothesize that the divergence of the viruses between the two locations is a recent event caused by host movement. More evolution models and more viruses complete genome sequences were needed to improve the understanding of WENV evolution.

The pathogenic potential of WENV to humans was the first reported in 2015 and remains an issue of further studies [8]. In Cambodia, the WENV was associated with human respiratory diseases. In this study, we have gained both molecular and serological evidence to demonstrate that brown rats (*R*. *norvegicus*) and oriental house rats (*R*. *tanezumi*) are the major natural host of WENVs. In Yunnan, these two rodent species are the dominant species and commensal with humans, suggesting a high risk of spillover of WENVs and occurrence of similar respiratory illness cases caused by WENV infection. Thus the ELISA method developed in this study can serve as a tool for screening WENV antibodies in undiagnosed fever patients. In Yunnan Province, the Haemorrhagic Fever with Renal Syndrome (HRFS) and Dengue fever are also occurring, which may lead to the misdiagnosis of the disease caused by WENVs or other mammarenaviruses. The ELISA test for WENV antibodies will reduce the likelihood of misdiagnosis, though the results need to be further verified by virus neutralization assay.

The recent report of WENV association with human illness reminds us there is still high risk of spillover of these rodent arenaviruses across species[22]. Moreover, we found that some rodent mammarenaviruses have a wider tissue tropism in their natural hosts. In addition to the liver, these viruses were also detected in the heart, spleen, lung, kidney, and intestine and may increase the spillover potential of some viral strains. Deep borderline, many remote areas, and abundant animal sources increase the burden to discover and control zoonotic diseases in Yunnan Province. Taken together, our results emphasize the importance of extensive surveillance of mammarenavirus in both their natural reservoirs and in humans.

## Supporting information

S1 Fig ELISA seroprevalence assay. A cutoff value of 0.03687 (horizontal line) was determined based on three times the OD values of negative controls. Samples with an OD value greater than the cutoff were deemed positive.
(TIF)

S2 Fig Western blot detection of viral antibodies in rat serum samples. Viral antibody positive (2016YM02, 2016YM41, 2016YM50, and 2015YM52) or negative (2016YM49 and 2013LX05) rodent serum samples were identified by ELISA, and tested against the viral NP by western blot. Human coronavirus (HCoV NL63) His-tagged NP were used as controls. Lanes: 1 and 2, negative control; lanes 3–6, ELISA positive sera (2016YM02, 2016YM41, 2016YM50, and 2016YM52); lanes 7, anti-His antibody.
(TIF)

S1 Table Mammarenavirus sero-detection results for small mammals collected in Yunnan Province. (DOC)
