# Supplementary figures and images for: Prevalence of Wēnzhōu virus in small mammals in Yunnan Province, China
Source: PLoS Negl Trop Dis. 2019 Feb 15;13(2):e0007049. doi: 10.1371/journal.pntd.0007049 (PMC6395006; doi:10.1371/journal.pntd.0007049)

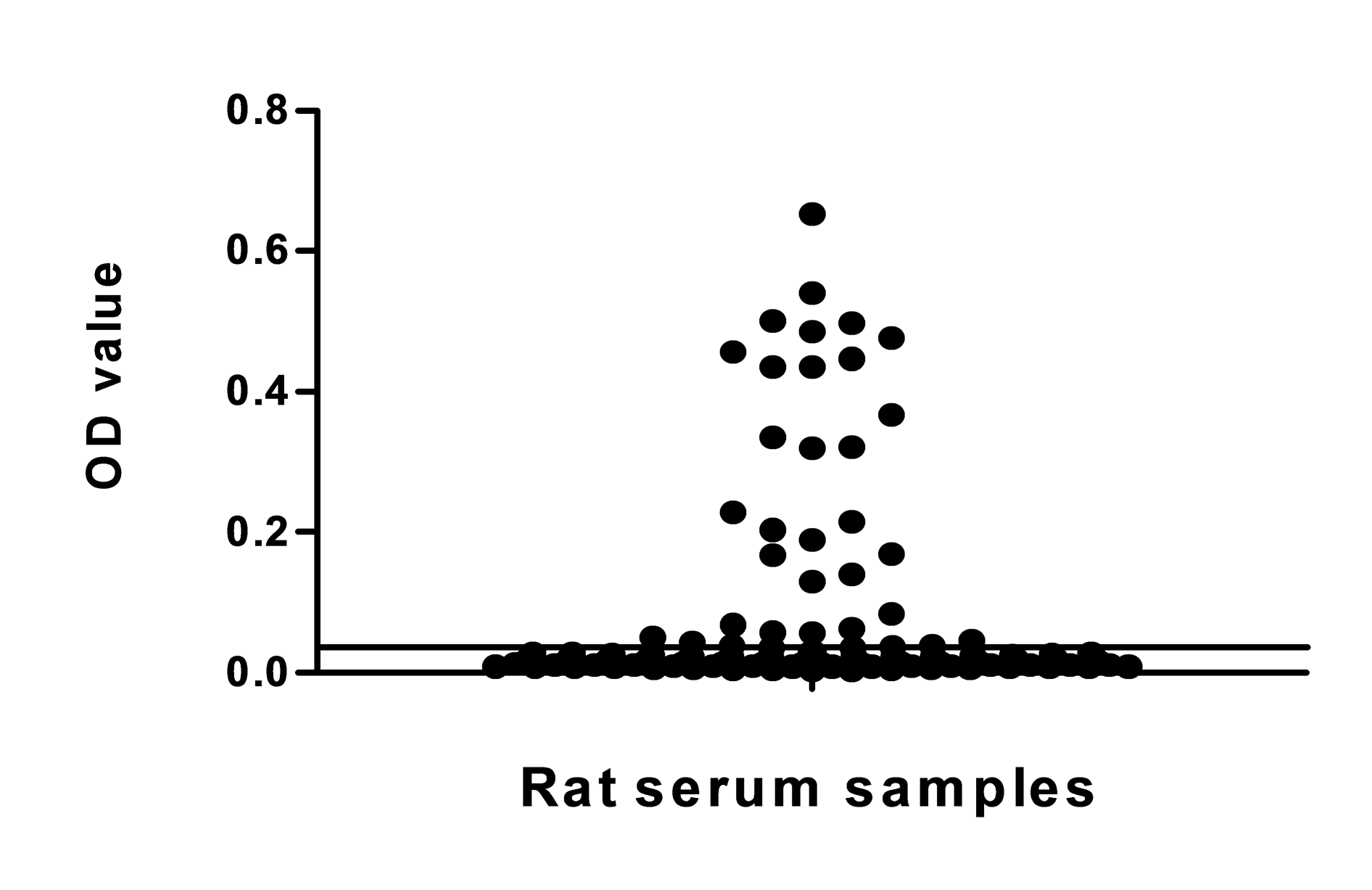

Supplement: S1 Fig — A cutoff value of 0.03687 (horizontal line) was determined based on three times the OD values of negative controls. Samples with an OD value greater than the cutoff were deemed positive. (TIF) [file pntd.0007049.s001.tif]

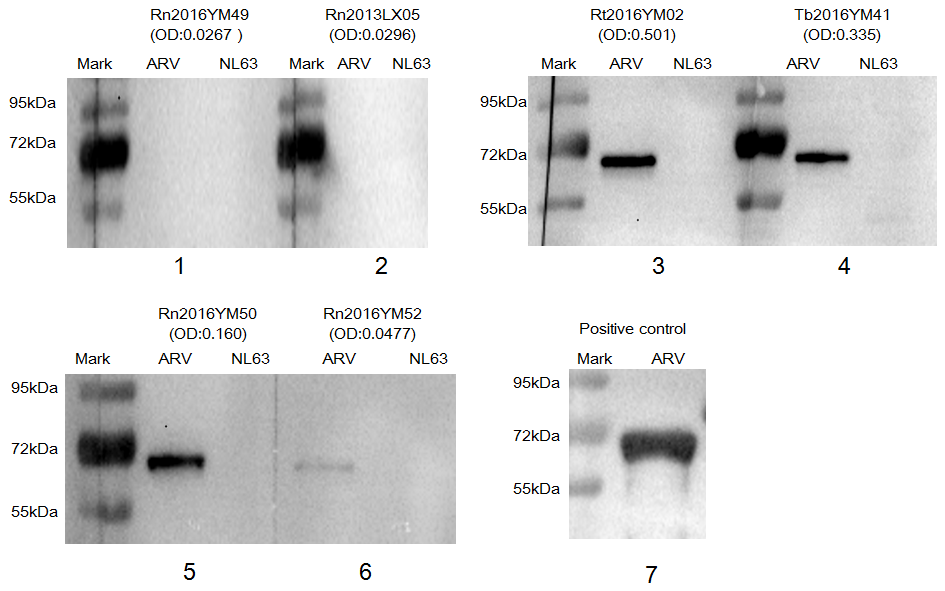

Supplement: S2 Fig — Viral antibody positive (2016YM02, 2016YM41, 2016YM50, and 2015YM52) or negative (2016YM49 and 2013LX05) rodent serum samples were identified by ELISA, and tested against the viral NP by western blot. Human coronavirus (HCoV NL63) His-tagged NP were used as controls. Lanes: 1 and 2, negative control; lanes 3–6, ELISA positive sera (2016YM02, 2016YM41, 2016YM50, and 2016YM52); lanes 7, anti-His antibody. (TIF) [file pntd.0007049.s002.tif]
