# Supplementary material for: Prevalence of Wēnzhōu virus in small mammals in Yunnan Province, China
Source: PLoS Negl Trop Dis. 2019 Feb 15;13(2):e0007049. doi: 10.1371/journal.pntd.0007049 (PMC6395006; doi:10.1371/journal.pntd.0007049)
Supplement: S1 Table — (DOC) [file pntd.0007049.s003.doc]

**S1 Table. Mammarenavirus sero-detection results for small mammals collected in Yunnan province.**

| Family | Genus | Species | Animal | Sampling site(s) (P/D *), year | | Total |
| --- | --- | --- | --- | --- | --- | --- |
|  |  |  |  | Luxi, 2013 | Yuanmou, 2017 |  |
| Muridae | Apodemus | *A. chevrieri* | Chevrier's field mouse | 0/16 | -- | 0/16 |
|  | Mus | *M. musculus* | Least Philippine Apomys | 0/30 | -- | 0/30 |
|  | Rattus | *R. norvegicus* | Brown Rat | 0/9 | 23/52 | 23/61 |
|  |  | *R. tanezumi* | Oriental House Rat | 0/2 | 1/1 | 1/3 |
| Mustelidae | Mustela | *M. sibirica* | Sibiriae montanis | 0/1 | -- | 0/1 |
| Soricidae | Anourosorex | *A. squamipes* | Chinese Mole Shrew | 0/5 |  | 0/5 |
|  | Crocidura | *C. attenuata* | Asian Gray Shrew | 0/1 | -- | 0/1 |
| Tupaiidae | Tupaia | *T. belangeri* | Northern Treeshrew | -- | 0/1 | 0/1 |
| Total |  |  |  | 0/64 | 24/54 | 24/118 |

*, P, positive number; D, detected sample number(s).
